# Burden level among parents of children with epilepsy

**DOI:** 10.1192/j.eurpsy.2022.816

**Published:** 2022-09-01

**Authors:** A. Guermazi, N. Charfi, M. Mnif, A. Zouari, W. Bouchaala, S. Ben Ncir, F. Kammoun, M. Maalej, C. Triki

**Affiliations:** 1 Hedi Chaker University Hospital, Sfax, Tunisia, Department Of Psychiatry C, Sfax, Tunisia; 2 Hedi Chaker University Hospital, Sfax, Tunisia, Child And Adolescent Neurology Department, Sfax, Tunisia; 3 Hedi Chaker University Hospital, Sfax, Tunisia, Child And Adolescent Neurology Department, sfax, Tunisia; 4 Faculty of medicine of Sfax, Lr19es15 Pediatric Neurology, sfax, Tunisia

**Keywords:** children with epilepsy, Zarit (ZBI-12), parents, Burden level

## Abstract

**Introduction:**

Family caregiving role of children with epilepsy involves managing the daily lives of these children with disabilities. This can lead to impaired physical and psychological health of the caregiver.

**Objectives:**

To assess the level of burden among caregivers of children with epilepsy and to determine the factors associated with it.

**Methods:**

It was a descriptive and analytical survey. It involved the caregivers of children with epilepsy who were admitted to the pediatric neurology department at the Hedi Chaker University Hospital in Sfax during the period from July to October 2020. We used the 12-item Zarit (ZBI-12), the State-Trait-Anxiety Inventory (STAI), and the “BECK” Depression Inventory (BDI-13) to assess caregiver burden, anxiety and depression respectively.

**Results:**

Forty-four caregivers participated in the survey. Their average age was 36 years and their relationship with patient was mother in 93.2% of cases. Among 44 children with epilepsy, 56.8% were boys and 34.1% were schooled. They had psychiatric comorbidity in 15.9% of cases. According to the ZBI scale, the level of burden was high in 45.5% of cases. The total ZBI score was significantly higher among caregivers with primary school level (p=0.05) and those with somatic disease (p=0.004). It was not correlated with the presence of child’s dependence on the others (p=0.20). High levels of depression, anxiety-state, and anxiety-trait among caregivers were correlated with the level of burden (p 0.000; 0.000 and 0.001, respectively).

**Conclusions:**

Being a caregiver of a child with epilepsy is a burdensome circumstance. Hence the importance of offering psychological assistance for these caregivers.

**Disclosure:**

No significant relationships.

